# Experiences With Unionization Among General Surgery Resident Physicians, Faculty, and Staff

**DOI:** 10.1001/jamanetworkopen.2024.21676

**Published:** 2024-07-17

**Authors:** Darci C. Foote, Audrey E. Rosenblatt, Daniela Amortegui, Carmen M. Diaz, Brian C. Brajcich, Cary Jo R. Schlick, Karl Y. Bilimoria, Yue-Yung Hu, Julie K. Johnson

**Affiliations:** 1 Department of Surgery, The Johns Hopkins University School of Medicine, Baltimore, Maryland; 2 Department of Surgery, Northwestern Quality Improvement, Research, and Education in Surgery, Northwestern University Feinberg School of Medicine, Chicago, Illinois; 3 Department of Anesthesia, Ann and Robert H. Lurie Children’s Hospital, Chicago, Illinois; 4 Department of Surgery, Surgical Outcomes and Quality Improvement Center, Indiana University School of Medicine, Indianapolis; 5 Division of Pediatric Surgery, Ann and Robert H. Lurie Children’s Hospital, Chicago, Illinois; 6 Now with Department of Surgery, University of North Carolina at Chapel Hill

## Abstract

**Importance:**

Labor unions are a mechanism for employee advocacy, but their role in surgery resident wellness is poorly characterized.

**Objective:**

To understand experiences with unionization among general surgery residents and residency program faculty and staff.

**Design, Setting, and Participants:**

This exploratory qualitative study included data from the Surgical Education Culture Optimization Through Targeted Interventions Based on National Comparative Data (SECOND) trial. In the exploratory phase of the SECOND trial (from March 6, 2019, to March 12, 2020), semistructured interviews about wellness were conducted with residents, faculty (attending physicians), and staff (program administrators) at 15 general surgery residency programs. Unionization was identified as an emergent theme in the interviews. Data analysis was performed from March 2019 to May 2023.

**Main Outcomes and Measures:**

The main outcome was resident and faculty experience with resident labor unions. In the qualitative analysis, lexical searches of interview transcripts identified content regarding resident labor unions. A codebook was developed inductively. Transcripts were coded by dyads, using a constant comparative approach, with differences reconciled by consensus.

**Results:**

A total of 22 interview transcripts were identified with relevant content. Of these, 19 were individual interviews conducted with residents (n = 10), faculty (n = 4), administrative staff (n = 1), a program director (n = 1), a department chair (n = 1), and designated institutional officials (n = 2), and 3 were from resident focus groups. Residents from all postgraduate year levels, including professional development (ie, research) years, were represented. Interviewees discussed resident unions at 2 programs (1 recently unionized and 1 with a decades-long history). Interviewees described the lack of voice and the lack of agency as drivers of unionization (“Residents…are trying to take control of their well-being”). Increased salary stipends and/or housing stipends were the most concretely identified union benefits. Unanticipated consequences of unionization were described by both residents and faculty, including (1) irrelevance of union-negotiated benefits to surgical residents, (2) paradoxical losses of surgery department–provided benefits, and (3) framing of resident-faculty relationships as adversarial. Union executives were noted to be nonphysician administrators whose participation in discussions about clinical education progression may increase the time and effort to remediate a resident and/or reduce educators’ will to meaningfully intervene. Active surgical resident participation within the union allows for an understanding of surgical trainees’ unique needs and reduced conflict.

**Conclusions and Relevance:**

In this qualitative study, unionization was a mechanism for resident voice and agency; the desire to unionize likely highlighted the lack of other such mechanisms in the training environment. However, these findings suggest that unionization may have had unintended consequences on benefits, flexibility, and teaching. Effective advocacy, whether within or outside the context of a union, was facilitated by participation from surgical residents. Future research should expand on this exploratory study by including a greater number of institutions and investigating the evolution of themes over time.

## Key Points

**Question:**

How do general surgery residents, faculty, and staff experience unionization?

**Findings:**

In this qualitative study including 19 health care professionals and 3 focus groups of residents at unionized general surgery programs, labor unions provided a mechanism for resident voice and agency, particularly regarding financial benefits; however, unintended consequences included a paradoxical loss of benefits and flexibility as well as resident-faculty conflict. Active representation helped increase the relevance of unions to surgical residents.

**Meaning:**

These findings suggest that voice and agency are essential components of resident wellness and can potentially be facilitated by resident labor unions with appropriate representation.

## Teaser

This qualitative study explores experiences with unionization among general surgery residents, faculty, and staff.

## Introduction

Since the Industrial Revolution, labor unions have represented employees and facilitated collective bargaining, advocating for improved employment terms and conditions. Workplace protections that are now foundational to US society—the federal minimum wage, Social Security, Medicare, and the 5-day/40-hour workweek—are attributable to unionization.^1^ In a union, employees pay dues to a third-party executive to negotiate on their behalf. The right of workers to form a union is protected by the federal National Labor Relations Act^2^ and is enforced by the National Labor Relations Board (NLRB), an independent federal agency. The role of the National Labor Relations Act is multifaceted and includes union election oversight and investigation of complaints for labor rights violations.^3^ Although federal law protects and regulates the steps to forming a union (Figure), the process can be contentious.

**Figure.**
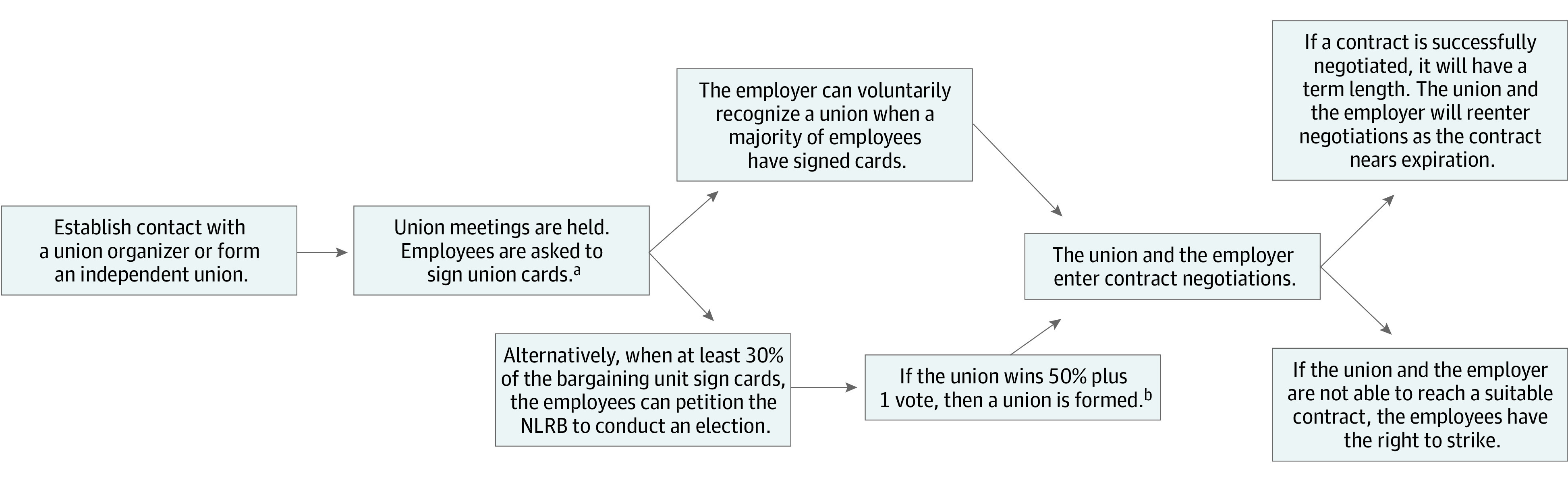
Step-by-Step Union Formation NLRB indicates National Labor Relations Board. ^a^Union cards are usually valid for 12 months. ^b^Refers to 50% of those who participate in the election (ie, does not need to be 50% of those employed).

Resident physician labor unions first appeared in 1934, when residents organized around compensation and working conditions as the Interne Council of Greater New York.^4^ In 1999, the NLRB issued a pivotal decision that physician residents are employees for purposes of federal labor laws.^5^ Resident physician unions usually include all residents across an organization as the same bargaining unit as they share a “community of interest,” regardless of medical specialty; residents must work together as a unified voice when negotiating via the union. Currently, an estimated 30 000 residents are unionized through the Committee of Interns and Residents, an affiliate of Service Employees International Union.^6,7^ Since 2020, the number of annual unionization movements has tripled, and an increasing number of programs are debating unionization.^6,8^

Resident unionization continues to be hotly debated.^9,10^ Recent survey data demonstrate that unionized programs are more likely to offer a housing stipend and more weeks of vacation time, but resident burnout, job satisfaction, and impressions of the educational environment are similar between surgical residents in unionized and nonunionized programs.^11^ The reasons for this similarity in wellness between unionized and nonunionized programs represent a gap in the literature. We therefore sought to describe experiences of residents, faculty (attending physicians), and staff (program administrators) with resident unionization using a qualitative approach.

## Methods

This qualitative study was approved by the Northwestern University Institutional Review Board. Participants provided verbal informed consent. The study followed the Standards for Reporting Qualitative Research (SRQR) reporting guideline.

### Site Visits and Interviews

 The Surgical Education Culture Optimization Through Targeted Interventions Based on National Comparative Data (SECOND) trial was a prospective cluster-randomized study of the impact of the learning environment on resident well-being.^12^ During the exploratory phase of the SECOND trial (between March 6, 2019, and March 12, 2020), 2-day site visits were performed at 15 general surgery programs. Site selection was previously described.^13^

During the 15 site visits, 366 individual interviews and 27 focus groups were conducted with residents, faculty, and staff. Each 45- to 60-minute semistructured interview was conducted by a pair containing at least 1 surgeon or surgical resident. Interviews were structured around a conceptual framework of surgical resident well-being but allowed for flexibility to promote candid conversations, given the wide scope of wellness.^14^ Follow-up questions were based on the progression of the conversation and impressions of the program over the course of the site visit, allowing for an in-depth understanding of the program context. Interviews were recorded, transcribed, and deidentified.

### Data Analysis

In our qualitative analysis, we identified unionization as an emergent theme. Lexical searches were performed to identify transcripts containing relevant content. The study team inductively constructed a codebook containing themes and subthemes. All transcripts were individually coded by dyads that included at least 1 surgeon and then were reconciled by consensus using a constant comparative approach in MAXQDA, version 2020 (VERBI Software GmbH).^15^ A secondary review of all data (by D.C.F., A.E.R., Y.-Y.H., and J.K.J.) confirmed that findings of the dyads were consistent. Data analysis was performed from March 2019 to May 2023.

## Results

We identified 22 transcripts with content relevant to unionization; 19 were individual interviews with residents (n = 10), faculty (n = 4), administrative staff (n = 1), a program director (n = 1), a department chair (n = 1), and designated institutional officials (DIOs) (n = 2) and 3 were from resident focus groups. Residents from all postgraduate years (PGYs) were represented, as were residents in their professional development years (hereinafter, research residents). All union-related interviews occurred at 2 programs: one that had recently unionized and another that had been unionized for decades. The following themes were represented in interviews from both institutions in our qualitative analysis: (1) surgical residents unionize for voice and agency, (2) union-negotiated benefits have varying levels of efficacy and relevance to surgical residents, and (3) unionization affects the educational environment.

### Theme 1: Surgical Residents Unionize for Voice and Agency

Surgical residents articulated that surgical culture is a barrier to voice, and the high workload is a barrier to advocating for improving the learning environment. Unionization allowed residents to reframe complaints and move into a proactive space of improving their working conditions, as noted by this PGY1 resident interviewee:

It’s a very surgical mentality to just plod through things and not complain. We have an [operating room] workroom where half the computers are always broken, there’s never toner in the computer, paper runs out. If one person just said to someone, “Can you fix this problem?” it would be fixed. But everybody just is like, “Oh, the computer is broken again, there’s no paper, [what a] hassle. Move somewhere else.” The union is kind of like the stand-in for that inertia that we have in our habits of just dealing with stuff.

All parties thus described unions as a unique mechanism for residents to voice concerns (Table), like this DIO:

**Table.** Representative Quotes About Resident Unionization

Theme	Quote (participant)
Theme 1: surgical residents unionize for voice and agency	“I think [resident unionization] was a good thing in many ways because we always wanted to help our residents.…I don’t think that they’re compensated enough, and I think they work 2 full-time jobs no matter how you do it, right? It’s 80 h, and…a very stressful period in their lives. So, we were really happy to have the union impose some things that people (the organization) would just have to come up with the money to do it.” (faculty)
Theme 2: union-negotiated benefits have varying efficacy and relevance to surgical residents	“We are contractually guaranteed paid…maternity and paternity leave.…Six weeks for a vaginal delivery, 8 for cesarean.…Paternity leave is 2 weeks.…Previously there was only a 4-day paternity leave. And we’ve kind of broadened out the term for primary caregiver and things like that.” (PGY5 resident)
	“The union is saying, ‘You get 4 weeks paid maternity in addition to your 4 weeks of vacation,’ but…the American Board of Surgery doesn’t allow that, so…what are you going to do? We have to follow ABS rules more than anything.” (PGY4 resident)
	“There’s this sentiment that everything needs to be standardized, so any benefits that surgery residents get, if the medicine residents don’t get them, they’re worried about a labor dispute, and so they’re saying that we can’t do party things.” (PGY5 resident 1)
	“Like our kickball, and stuff is no longer going to be allowed to be paid for by the program.…We used to get a better education stipend than the other programs, which is now being taken away, and more food money than some of the other programs, which is now being taken away.” (PGY5 resident 2)
	“I guess maybe we should have cared more when this all happened.” (PGY5 resident 1)
	“That’s what it really comes down to, we had opportunities to be involved and didn’t.” (PGY5 resident 2)
Theme 3: unionization affects the educational environment	“There’s been a significant uptick in union activity.…Most unions started working very aggressively to demonstrate value to their members, and the way you have to demonstrate value is showing that…the employer is doing things that only the union can protect you from.…We had grievances filed at approximately a rate of 1 a month.” (DIO)
	“So, it has a lot to do with who’s leading it. Not necessarily from the resident’s side, but…because it’s an independent organization, they hire essentially an executive to run it. And the person that’s in that seat now is actually out of the [manufacturing] industry, which, as you can imagine, brings a culture of conflict and persecution to it a little bit. So, it’s like: grievance, grievance, grievance, grievance. Everything is viewed from a conflict lens, and so literally there was a point where this person had 200 grievances filed against the GME office and the hospital.” (program director)
	“And they’ve been largely overreaching lately where they’re trying to insinuate themselves into basically academic issues about our residents’ performance. Basically, it’s turning into a little bit of the nursing union concept, where it’s, ‘You cannot fire me. No matter what I do, you can’t possibly fire me because I’ve got the union rep right next to me.’” (program director)
	“We have actively worked around the issues of academic due process with this union because…they challenged the program director’s authority and responsibility for signing off on competency at the end of training. They said that if there’s academic discipline that results in an adverse outcome like probation or dismissal from a program, that those decisions needed to go to binding arbitration by someone who’s not in the profession, which, of course, really flies in the face of what we try to do in training and what we’re mandated to do.” (DIO)
	“There’s a lot of fear from the hospital.…They’re worried about labor disputes if somebody feels like a contract is violated.” (senior resident, focus group PGY 3-5)

Abbreviations: ABS, American Board of Surgery; DIO, designated institutional official; GME, graduate medical education; PGY, postgraduate year.

I think that [the union] provide[s] a communication mechanism that is not present in other places.

###  Theme 2: Union-Negotiated Benefits Have Varying Efficacy and/or Relevance to Surgical Residents

Residents and faculty both enumerated financial gains negotiated by the union. These were primarily achieved through increased salary, as described by a PGY5 resident:

We are paid very well, which I would argue is a wellness thing. We’re paid probably $10,000-$15,000 more than our closest neighbor.…We have great benefits in terms of our health insurance.…Overall, I think compared to other institutions,…we’re treated much better from the union’s standpoint.

Other financial gains attributed to union bargaining included meal, housing, and educational stipends; subsidized parking; medical equipment (eg, loupes); licensing or board examination registration fees; and fertility coverage (eg, oocyte cryopreservation). These benefits were noted to provide substantial stress reductions, as stated by a research resident:

Living in [this city] can be expensive. That’s probably been the biggest stressor in residency, especially intern year. Since intern year, it’s changed dramatically with us getting unionized.…I was seriously living paycheck-to-paycheck the first 2 years. So, things have changed for the better in that sense.

Although financial benefits were universally appreciated, surgical residents often found other union-negotiated benefits to be irrelevant or inaccessible to them. In both programs, surgical residents reported that they could not utilize union-negotiated time-off provisions: they could not take full parental leave without extending their training due to American Board of Surgery requirements (Table), they could not take guaranteed holidays because there was inadequate clinical coverage, and they did not use preventive care time because surgical culture prizes self-sacrifice. One PGY5 resident stated the following:

Our contract guarantees us 6 hours of preventive care, but none of us collect on that because we’re afraid to collect on it.…Maybe afraid isn’t the right word, but you just don’t look dedicated.

Moreover, unions create rules and regulations to enforce uniformity across programs, which could result in 2 types of loss. The first was a loss of previously established benefits (presumably because other, less well-funded departments could not match the resources of surgical departments), as indicated by a faculty member:

We are not going to be able to offer anything that is over and above what the contract specifies.…We used to give them $1000 for academic accounts and things to spend, so, we’re not able to give that money anymore. So, it has to be whatever everybody else gets, whatever gets negotiated.

The second was a loss of flexibility for the program to meet residents’ individual needs, as noted by 2 PGY5 residents:

We take pride in making the schedule in a way that’s good for resident well-being. All of that work, to have the possibility that these random, new restrictions are going to be put on it, after we’ve spent a lot of time accommodating every request we’ve ever gotten for the schedules.

I get a little upset about this idea that Thanksgiving, Christmas, and New Years [are union holidays] because I don’t celebrate Christmas, but my Hindu holidays, I don’t have off.

The previous quote speaks to how standardization for the benefit of the majority may unintentionally result in exclusionary practices.

Residents acknowledged that lack of surgical representation within the union structure was a major reason that union-negotiated benefits were of limited utility to the surgical subspecialties (Table). One PGY4 resident stated the following:

A lot of the potential benefits are realistically probably not going to be reaped by surgical subspecialties.…Admittedly, could we have been involved in it infinitely more than 99% of us have been? Yes.

###  Theme 3: Unionization Affects the Educational Environment

Educational leadership noted that because unions are a third party whose role is employee advocacy, they may amplify conflict to demonstrate value to their membership (Table). Leaders perceived that union executives encouraged residents to engage in conflict rather than bring issues directly to their leaders to be solved collaboratively. In the words of a program director:

Unfortunately, I think [the union] is a negative influencer on resident culture, because [the residents] sort of hear from this person, like, ‘You let me know the first sign of trouble—I’ll take care of it.’ And you know, it creates this suspicion, this mistrust that everybody’s out to get them.…That’s a unique challenge we have here…that independent union stance that tends to inject conflict into it.…So that is a challenge, even for me, in a program that functions in a pretty healthy way because residents, if they’re having an issue, there’s probably a 2-to-1 chance they’re going to go to the union before they go to me.…That’s not always the best way to solve problems; it tends to make problems much bigger than they needed to be.

This framing of the union as the sole recourse for residents against an unsupportive administration was perceived to be further influenced by hired union executives’ prior experiences in other industries (Table).

When surgical residents participated within the union, educational leadership complimented these resident leaders for their ability to mediate conflict between the union and the institution. Two interviewees—the first a program director, the second a DIO—offered the following:

One of our residents is…the [leader] of the [union] this year. And she’s a very rational person that understands which fights are worth having and which ones aren’t.

We had two pretty contentious [resident leaders] of the union.…The [resident leaders] of the union are elected by the house officers, and the current [resident leader] is actually a general surgeon, and since she has taken the post, we’ve only had 1 grievance, which was subsequently dropped by them.

As reported by faculty, administrators, and residents, conflict extended beyond specific union-filed grievances to affect relationships between residents and their faculty. Two more interviewees—the first a PGY5 resident, the second a DIO—stated the following:

It violates the educational contract we have with our faculty.

Unfortunately, the process of unionizing puts us in an adversarial relationship with residents. That’s just the nature of the beast.…The nature of unionizing is not all that compatible with mentoring and thinking about the members of the union as learners.

Conflicts around clinical educational assessments were particularly contentious. Residents described a sense of employment protection from having hired union executives participate in deliberations about remediation, probation, or dismissal; they perceived that this process ensured fair due process, as reflected by a PGY5 resident:

If there’s…disciplinary action, I’ve heard from people that the union really sticks up for you, and so I think…we’re mostly very pro-union.

However, educational leadership felt that because union executives are nonclinicians, their involvement in discussions of academic advancement was inappropriate and hindered necessary remediation efforts, ultimately affecting training quality and patient safety (Table). One DIO described this as follows:

Across the country, I think a number of DIOs have raised a concern that unions, which are organized around employment issues, are now trying to make forays into academic issues. And I will tell you that the institutions feel pretty strongly…that the programs actually have jurisdiction over who advances, who graduates.…The programs…the institutions feel that they should have sole direction over what patient duties are assigned.…We feel that we own the public trust, which is not to put people in positions of unsupervised activity, whether it be graduating them or advancing them, that we…don’t feel is appropriate.…We protect the public. We’re paid for that, that’s what we do: we try to graduate safe, competent…physicians.

Aside from its effect on resident-faculty relationships, unionization was described as having positively affected the clinical and educational environment by advocating for increased support staffing. As a program director noted:

And through the years, [the union has] served different roles. The fundamental purpose for its existence is to bargain collectively with the hospital every 3 years or so. [Earlier,] it was mostly around what your salary’s going to be, making sure you have parking, making sure you have meal tickets, that sort of mechanistic stuff. And it changed a little bit after that—[the union] worries more about getting the hospital to invest in better support resources, like we need more radiology techs at night or need more phlebotomy teams. So, it’s still very constructive.

This quote speaks to the evolution in negotiations as the union at an institution matures and the potential of union-led advocacy to benefit patient care as well as resident working conditions.

## Discussion

This study of the experience of resident unionization at 2 institutions highlights the important role of voice (an expression of a wish, choice, or opinion) and agency (the capacity to act or exert power and influence) in resident wellness. Voice and agency are critical to employee engagement and well-being across work environments.^16^ They are prerequisites for the assertion of autonomy, a key element of physician wellness.^14,16,17,18,19^ Bongiovanni et al,^20^ in a qualitative study of residents who left general surgery, identified lack of voice as a major reason for attrition. Our data provide insight into how leaders, departments, and institutions may effectively advocate on behalf of their residents and, more specifically, how they may do so within the context of resident unionization.

Freeman and Medoff^21^ described 2 mechanisms of combating workplace problems: exit or voice. However, because residency is largely designed to be started and completed within the same institution, residents have limited options for exit; indeed, only 20% of general surgery residents who leave their residency program switch to another general surgery program.^22^ Because residents depend on their employer to provide the training they need to progress in their careers,^23^ concerns about the lack of exit options may precipitate reservations about expressing voice. In this study, residents spoke about how unionization provided a mechanism for raising and addressing issues. Such representation has been argued to provide an avenue for promoting meaning in work and life.^24^

Interviewees particularly valued their union-negotiated financial benefits. The importance of this benefit cannot be overstated, particularly in the context of stagnant resident wages relative to inflation^25,26^; financial security ranks second in Maslow’s hierarchy of needs. Notably, no resident mentioned the cost of union dues. In our prior study of resident unions, using the American Medical Association FRIEDA database, we found that resident salaries were similar between unionized and nonunionized programs, after adjusting for local cost of living.^11^ Although often cited as evidence that unionization has no effect on resident benefits or well-being, the current data suggest a possible alternative explanation: programs that are behind their local or national peers are more likely to unionize, allowing them to catch up to, rather than surpass, their peer institutions.^27^ Thus, residents may be accurately perceiving a financial benefit compared with their preunionization state, rather than compared with their peers. Importantly, union advocacy was reported to benefit patients as well as residents, providing an effective potential counterpoint to the criticism that resident unionization compromises patient care.^28^

Both residents and faculty described unionization as negatively affecting residents’ relationships with their faculty. Krzyzaniak et al^29^ recently explored this detrimental outcome on a particularly important faculty-resident relationship—the program director—and provided suggestions for mitigating negative consequences. The approach of the union executive may greatly influence the tone of negotiations and the general sense of resident-faculty collegiality. To be effective, resident union executives must fully understand residents’ dual roles as learners and employees. Educational leadership specifically reported challenges to their ability to remediate residents. Regardless of unionization status, active support of the resident-faculty relationship, emphasizing the mutual goal of training competent surgeons, by residents, faculty, educational leadership, and the union alike, is critical to both the education and well-being of residents. As a field, we must acknowledge that biases in our current evaluation methods contribute to known racial, ethnic, and gender disparities in autonomy and milestone attainment.^30,31,32^

Finally, our findings suggest the need for surgery residents to be active participants in their unions to ensure that their unique needs are considered. Representation is necessary to ensure that any third-party union executive understands all employees’ needs. Surgical residents may have different goals than their nonsurgical peers, and it is not possible to represent their voice or enact agency on their behalf in their absence. Participation may mitigate many of the negative experiences with unionization, such as irrelevant or inaccessible benefits and losses in flexibility or resources for surgery residents. Active surgical resident participation within the union was noted to reduce conflict between the union and the institution.

### Limitations

This study has several limitations. First, although we visited 15 programs, unions were discussed in only 2. However, these 2 institutions reflect a wide range in longevity of experience with unions, and we identified consistent themes across sites. The difference in union maturity of the institutions and participants included in our study indicates the themes we identify endure over time. Additionally, the drivers of unionization identified in this article (ie, the desire for voice and agency) were identified in programs not included in this analysis, indicating a universality in themes.^17^ Second, interviews were structured around a general wellness framework and were not specifically designed to probe into experience with unions; indeed, unionization was itself an emergent theme. This methodology was intentionally designed to investigate wellness, which is necessarily defined by the individual, allowing discussions to be organically driven by interviewees. This limitation is also a strength, as we did not ask proscriptive questions around unionization that could have influenced participant responses; rather, participants volunteered their perspectives around unions freely. However, it is possible that other themes may have emerged with more structured questions around unionization. Third, nonunionized programs’ opinions on unionization were not included. Fourth, interviews were conducted before the COVID-19 pandemic, after which resident unionization has risen.^33,34^ However, the ongoing discussions around unionization since 2019 suggest that our themes are increasingly relevant.^6,24,29,33^ Fifth, perhaps due to the timing of the study, we did not identify any programs that were in the process of unionizing. Sixth, residents discussed their benefits, but we did not attempt to verify these benefits. However, it is the experience of the individual that is relevant to this research, rather than the veracity of the contractual details. Finally, one subtheme—the evolution of the union to affect the clinical environment—occurred with union maturation and, thus, was supported by only a single institution. Future work including a greater number of institutions and experiences, incorporating the effects of the COVID-19 pandemic, and confirming described benefits over time would be expected to expand on our findings and likely identify other salient themes.

## Conclusions

In this qualitative study, residents sought unionization as a means for agency and voice. These findings suggest that although unionization provides some benefits, it may have unintended and sometimes paradoxical consequences. Surgical resident representation is critical to ensuring meaningful advocacy. Future research should expand on this exploratory study by including a greater number of institutions and investigating the evolution of themes over time.

## Notes

Supplement.
Data Sharing Statement
