# Supplementary material for: Experiences With Unionization Among General Surgery Resident Physicians, Faculty, and Staff
Source: JAMA Netw Open. 2024 Jul 17;7(7):e2421676. doi: 10.1001/jamanetworkopen.2024.21676 (PMC11255910; doi:10.1001/jamanetworkopen.2024.21676)
Supplement: Supplement. — Data Sharing Statement [file jamanetwopen-e2421676-s001.pdf]

Data Sharing Statement

Foote. Experiences With Unionization Among General Surgery Resident Physicians, Faculty, and Staff. *JAMA Netw Open*. Published July 17, 2024.
doi:10.1001/jamanetworkopen.2024.21676

Data
Data available: No
